# Research on the Impact of Enterprise Green Development Behavior: A Meta-Analytic Approach

**DOI:** 10.3390/bs12020035

**Published:** 2022-02-03

**Authors:** Xingwei Li, Jiachi Dai, Jingru Li, Jinrong He, Xiang Liu, Yicheng Huang, Qiong Shen

**Affiliations:** College of Architecture and Urban-Rural Planning, Sichuan Agricultural University, Chengdu 611830, China; xwl@sicau.edu.cn (X.L.); 2020325016@stu.sicau.edu.cn (J.D.); 2021325022@stu.sicau.edu.cn (J.L.); hejinrong@stu.sicau.edu.cn (J.H.); liuxiang@stu.sicau.edu.cn (X.L.); huangyicheng@stu.sicau.edu.cn (Y.H.)

**Keywords:** green development behavior, green supply chain management practice, cleaner production, meta-analysis, organizational behavior, industrial ecology

## Abstract

The environmental situation is not optimistic. Improving the level of enterprise green development behavior can help enterprises to comply with the trend of environmental protection. However, existing studies do not explain the factors influencing enterprise green development behavior. This research collects and screens 33 empirical studies related to enterprise green development behavior from multiple authoritative data platforms, which cover 10 different countries and regions. A quantitative approach is then used to comprehensively explore the influencing factors, deeply dig into their degree of influence, and explore the moderating effect of the moderators. The results show the following: (1) corporate tangible resources, corporate intangible resources, market environment, policy and institutional environment, and public supervision have positive effects on enterprise green development behavior, and there are differences in the degree of influence; (2) corporate intangible resources have the most significant influence on enterprise green development behavior; (3) the size, region, and industry of enterprise can moderate enterprise green development behavior. This research suggests four participants: society, enterprise, market, and government. The research results are intended to provide a basis for researchers to further study enterprise green development behavior for specific industries and promote enterprise green development.

## 1. Introduction

Since the industrial revolution, enterprises have adopted a production model with high resource consumption and high turnover, which has increased the pressure on the ecological environment. To coordinate the relationship between economic development and ecological environment protection, the concept of green development came into being [1]. China [2], the UK [3], and Japan [4], among others, are actively promoting green development to achieve a comprehensive green transformation of economic and social development. Green development has now been adopted by many industries, including construction and finance, and has become a global consensus [5]. Total global carbon emissions in 2020 were 5.15% lower than in 2019 [6]. In the context of economic globalization, it is extremely urgent for enterprises to practice green and low-carbon development and achieve green transformation. According to the literature [7,8,9], enterprise green development behavior (EGDB) in this study is referred to as behaviors that seek to achieve their own green development goals, where enterprises adopt an organizational behavior that is conducive to both environmental protection and enterprise development. As an integral part of green development practices, EGDB receives attention from participants such as government, society, and enterprise decision makers—each playing an active role in addressing environmental issues. For example, to improve the level of enterprise green development in various fields (traditional industries and emerging e-commerce industries, etc.), the Chinese government has made efforts to create a fair competitive market environment by improving environmental and economic policy measures. The Chinese government has also incorporated the issue of combating environmental pollution into its national economic and social development plans (e.g., the 13th Five-Year Plan and the 14th Five-Year Plan). Non-governmental organizations (NGOs) play the role of assisting in monitoring and enforcing the law in the process of environmental issues [10]. From the perspective of enterprise organizations, different degrees of green preferences among enterprise decision makers can also make a difference in the extent of EGDB. It is not difficult to find that, in a realistic background, EGDB as an organizational behavior is generally influenced by multiple factors inside and outside the organization. Although some studies have revealed the influencing factors of EGDB [11,12], there is a lack of systematically revealing and validating the influencing factors and the degree of influence of EGDB.

Theoretically, the field of enterprise green development is flourishing. Academia has conducted a series of relevant studies and verifications on green development of enterprises from different industries, such as industry [13] and manufacturing industry [14], from different perspectives, such as environmental regulation [15] and the green tendency of organizational decision makers [16]. Although existing studies have demonstrated the influencing factors of enterprise green development in different industries, most of them focus on one-sided aspects, such as government environmental regulation, to study influencing factors of enterprise green development. It cannot fundamentally reveal the factors that affect EGDB in a general sense. Therefore, unlike previous studies [11,12], this study constructs and verifies a theoretical model of the factors influencing EGDB, and systematically sorted out the empirical research related to EGDB using meta-analysis methods. This research attempts to answer the following scientific questions: What are the influencing factors of EGDB? What is the corresponding degree of influence? Can different moderating variables play a moderating role in EGDB and its influencing factors? How can the level of EGDB be improved?

The innovation of this research is that it introduces meta-analysis into the research field of EGDB, which provides a new research idea for enhancing the level of EGDB. In addition, this research integrates the research on the influencing factors of EGDB from multiple scenarios and perspectives, which further expands the research scope of EGDB and reveals a new theoretical model of EGDB. Based on 33 empirical studies of different scenarios, published in global academic journals from 2006 to 2021, this research uses meta-analysis to construct and verify the influencing factors model of EGDB. Furthermore, this research explores potential moderating variables. As a quantitative research method, meta-analysis extracts reliable information from existing literature for analysis. This method has a high degree of objectivity, which makes the research conclusion more comprehensive and reliable. By looking into the relevant research on the existing literature of EGDB, it was found that previous researchers chose a single research object and mostly focused on industrial enterprises. Therefore, meta-analysis is urgently needed to systematically integrate the existing research on EGDB. Moreover, this study expands the research in the field of green development and organizational behavior to provide detailed suggestions and guidance for enterprise managers when they practice green development behavior. It also provides an effective reference for the government to make guiding policies on the green development of enterprises.

## 2. Theoretical Basis and Model Construction

Li believes that the main problem facing green development at present is the lack of top-level design of governance to guide and coordinate the green development behaviors of all subjects [17]. Meanwhile, the researchers found, during the literature search, that the research literature on EGDB is still scarce. Fortunately, the GDBP–IE (green development behavior and performance of industry enterprise) theory explains the influence factors on the relationship between EGDB and performance, which was proposed and validated by Li et al. [7]. The GDBP–IE theory states that, to achieve their own green development goals, industrial enterprises exist in organizations in the form of green development behaviors. In practice, industrial enterprises will form two specific organizational behaviors—green supply chain management practices (GSCMP) and cleaner production behaviors (CPB)—to achieve green development of enterprises [7]. This type of behavior is a green behavior at the organizational level, and is closely related to people’s food, clothing, housing, and transportation [18]. Compared with agriculture and service industries, the types of enterprises involved in the production and operation of industrial enterprises are more complex. Therefore, this study adopts the GDBP–IE theory.

Based on the GDBP–IE theory, the influencing factors of EGDB can be divided into two levels: internal influencing factors and external influencing factors [8]. The internal influencing factors include intangible resources and tangible resources. The external influencing factors of enterprises include market environment, public supervision, and policy and institutional environments.

### 2.1. Corporate Intangible Resources (CIR) and Corporate Tangible Resources (CTR)

Resource-based theory states that both tangible and intangible resources are business resources. Corporate tangible resources are used by enterprises as resources for producing output and earning profits, including financial capital, manufacturing capital, and natural capital used for the operation of the organization [19]. Enterprises usually weigh their costs and benefits before deciding. Enterprises choose to implement only when the costs of implementation are less than the benefits of implementation [19,20]. Therefore, this research proposes the following hypothesis:

**Hypothesis** **1** **(H1).** *CTR has a positive and significant influence on EGDB.*

Contrary to corporate tangible resources, corporate intangible resources are non-physical resources, which play a key role in sustainable competitive advantage [21]. Oprean-Stan et al. [22] divide corporate intangible resources into two levels—knowledge resources, at the individual level, and intangible resources, at the organizational level. Based on the strategic layout of the enterprise formed by the combination of the enterprise employees’ knowledge resources and the enterprise organizational resource, innovative management strategies [23] and other measures are adopted to promote EGDB. In addition, as the core of the organization, the background and experience of the senior managers of the enterprise will drive EGDB [24]. Therefore, this research proposes the following hypothesis:

**Hypothesis** **2** **(H2).** *CIR has a positive and significant influence on EGDB.*

### 2.2. Market Environment (ME)

Enterprises are always in a market environment characterized by consumer purchasing preferences, competition among peer enterprises, and cooperation between upstream and downstream enterprises in the supply chain. In addition, the decision making of enterprises also needs to be based on certain market conditions, and it is impractical to make decisions based on specific circumstances [25]. Due to the consideration of environmental and economic benefits, the market positioning of enterprises can promote cleaner production behavior [26]. Under the pressure of customers’ demand for green products, manufacturers have to coordinate their environmental goals with development strategies [27]. Therefore, this research proposes the following hypothesis:

**Hypothesis** **3** **(H3).** *ME has a positive and significant influence on EGDB.*

### 2.3. Public Supervision (PS)

Public supervision is mainly enacted by residents, the media, and NGOs, etc. The theory of environmental public believes that citizens have the right to monitor social environmental resources, as they are the common property of all citizens. The public has achieved the effect of supervising the environmental behavior of enterprises by exerting indirect pressure on them [28]. Zhao [29] believes that the participation of public supervision on environmental protection has a significant impact on the implementation of green development behavior by enterprises in the region. Therefore, this research proposes the following hypothesis:

**Hypothesis** **4** **(H4).** *PS has a positive and significant influence on EGDB.*

### 2.4. Policy and Institutional Environment (PIE)

To reduce environmental pollution problems, governments are actively introducing policies to guide enterprises to improve their green behavior. Furthermore, some enterprises with foreign trade businesses need to comply with local environmental requirements and regulations, such as through attaining ISO14001 environmental certification [30]. Enterprises are the main target subjects of environmental regulation, and the government can indicate the direction of R&D for enterprise innovation through environmental regulation [31]. Therefore, this research proposes the following hypothesis:

**Hypothesis** **5** **(H5).** *PIE has a positive and significant influence on EGDB.*

### 2.5. Moderator

In the meta-analysis, as the third variable, the moderator affects the direction or strength of the relationship between EGDB (independent variable) and the influencing factors of EGDB (dependent variable) [32]. The moderators in meta-analyses are usually extracted from the control variables in the empirical study [33]. Considering that larger enterprises have more resources than small-scale enterprises, there may be situations where the enterprise size can directly affect the results of the implementation of EGDB [34]. The economic development level of the selected sample object and the inconsistency of the industry will also affect the practice results of green supply chain management [35]. Therefore, the moderators are determined by the size, region, and industry of the enterprise; the following hypotheses are put forward:

**Hypothesis** **6** **(H6).** *Firm size plays a moderating role between EGDB and its influencing factors.*

**Hypothesis** **7** **(H7).** *The region where the enterprise is located plays a moderating role between EGDB and its influencing factors.*

**Hypothesis** **8** **(H8).** *The industry in which the enterprise is located plays a moderating role between EGDB and its influencing factors.*

**Figure 1:**
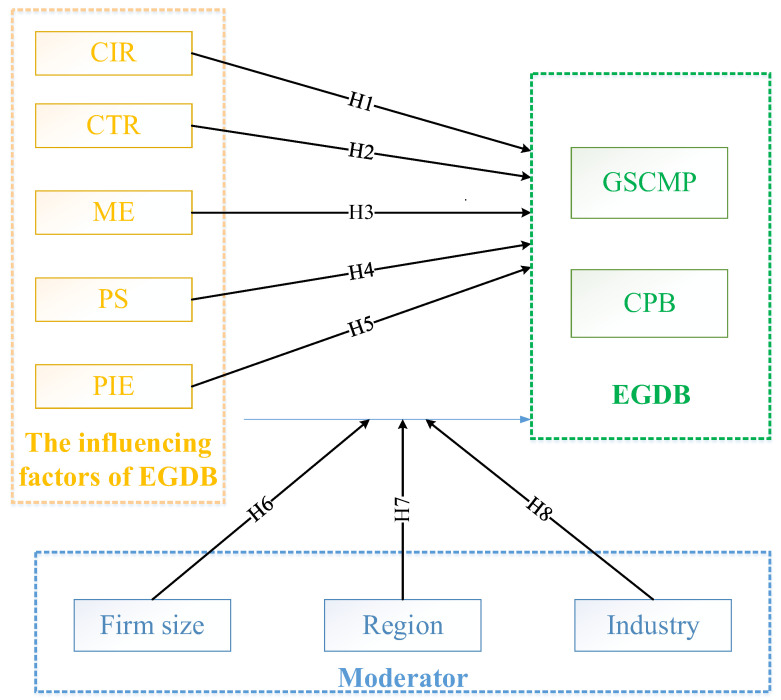
The research framework.

The research framework is shown in Figure 1 to guide the subsequent research process based on the above discussion.

## 3. Materials and Methods

### 3.1. Meta-Analysis

The classical definition of the meta-analysis by Glass is the statistical analysis of a large number of analyses from individual studies to integrate the results of the studies [36]. Contrary to traditional methods, a meta-analysis uses more objective data collection and numerical analysis. Existing quantitative studies on EGDB influencing factors meet the basic requirements of meta-analysis, based on the amount of empirical literature.

### 3.2. Retrieval Strategy

To collect related research on EGDB, GSCMP, and CPB, this study took December 2020 as the cut-off point and retrieved the literature published before the cut-off point. We use the electronic data platforms Science Direct, Web of Science, Springer, Google scholar, Wiley, IEEE, ProQuest Dissertations, and Theses Global database, using “enterprise green development behavior”, “firm green development behavior”, “company green development behavior”, “ corporate green development behavior”, “green supply chain management practice”, and “cleaner production behavior” as keywords to search for related journal articles—determining that each keyword must appear in the title or abstract of the complete field. The retrieved results found that the number of target literature retrieved was insufficient to meet the study criteria due to the newness of the field. Therefore, keywords such as “green development behavior”, “green supply chain management”, and “cleaner production” were searched, based on saving these documents, and appeared as a complete field in the title or abstract. To reduce the study’s bias, the researchers used the same search method, adding more studies over a time span from December 2020 to May 2021. So, as of 6 June 2021, the researchers had collected a total of 1677 articles.

### 3.3. Sample Screening

Since the collected studies include repetitive studies that can be searched in multiple databases, duplicate studies were eliminated, leaving 1373 articles.

### 3.4. Inclusion Criteria

To screen out the samples needed for meta-analysis, based on discussions between the authoritative literature and researchers on multiple meta-analysis methods, the following inclusion criteria were formulated: (1) research objects are enterprises; (2) research contents include the influencing factors of EGDB, green supply chain management, GSCMP, sustainable supply chain management, CPB, or cleaner production; (3) the research form is empirical research, excluding literature reviews and case studies; (4) studies must report the effect size (Pearson correlation coefficient r, Cohen’s d, Fisher’s z, or Hedge’s g, etc.) and sample size.

**Figure 2:**
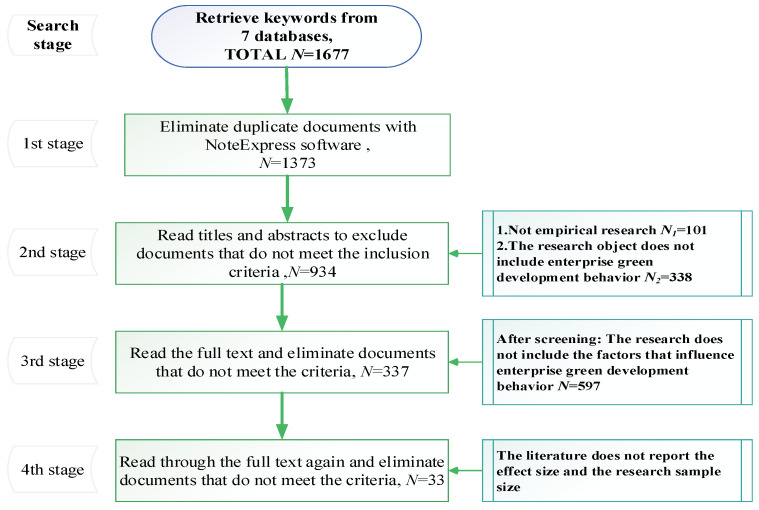
Selection process.

First, the researchers read the title and abstract of each study, excluding literature reviews and studies that were not company-specific, leaving a total of 337 studies. Then, the researchers went through the full text of the 337 studies, excluding studies that did not include factors affecting EGDB and those that did not report effect sizes and sample sizes, leaving 33 studies at the end. The specific selection process is shown in Figure 2.

### 3.5. Studies Code

To extract the various data required for the final target studies, the researchers coded the final target studies. Due to a large amount of data extraction, and to avoid coding errors, this study used a predesigned data extraction table, to have relevant data extracted by one researcher and have it reviewed by another researcher.

The effect size can reflect the importance of the effect and is the most frequently applied research indicator in meta-analyses [37]. During the extraction of the data, the effect sizes were found to be mostly described by correlation coefficients. Since the correlation coefficient r is not an equidistant scale, it does not simply average. Therefore, Fisher’s z was used as the effect size in the research. Based on the results of Borenstein et al. [37], the remaining effect sizes from all the included studies were subjected to Fisher’s z transformation. Then, the obtained Fisher’s z was taken as the final effect value, and the calculation formula of the conversion is shown in Formula (1), as follows:

(1) $$z=0.5\times \ln\left(\frac{1+r}{1-r}\right)$$

The encoding table is shown in Table 1. In the research, CMA 3.0 (Comprehensive Meta-Analysis V3 Software) was used for the meta-analysis, and the data in the coding table was entered CMA 3.0.

## 4. Analysis and Results

To better display the results of the meta-analysis, the researchers divided the results into six parts: bias test, heterogeneity test, outlier test, sensitivity analysis, main effect test, and moderator test.

### 4.1. Bias Test

Due to the existence of gray literature, unpublished literature, and non-English language literature, etc. [65], the ideal situation that all the literature meeting the criteria is included in the study was not possible in the realization process.

**Figure 3:**
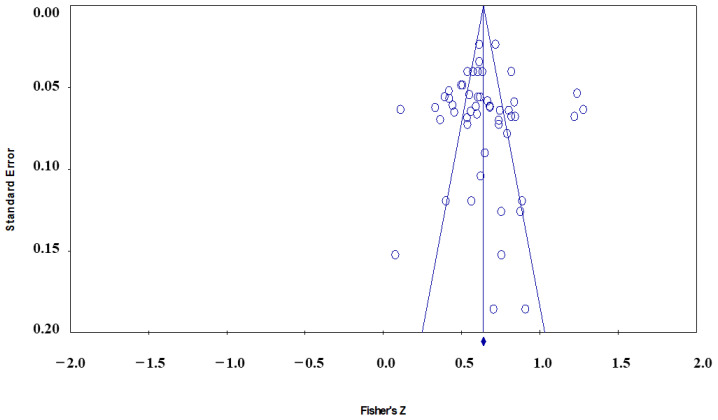
The funnel plot of the whole sample.

According to the funnel plot Figure 3, it can be observed that the research literature is not completely symmetrically distributed on both sides of the total effect size, which indicates that the influencing factors of EGDB and EGDB may be biased. However, the funnel plot is only a preliminary check for publication bias from a subjective point of view. Next, Rosenthal’s fail-safe N, the Begg and Mazumdar rank correlation, and Egger’s regression intercept are required for more accurate bias tests.

The publication bias test results obtained by CMA 3.0 software are shown in Table 2. In Rosenthal’s fail-safe number, when α = 0.05, z-value > 1.96, *p* < 0.001—that is, there is no bias in this study. In the Begg and Mazumdar rank correlation, *p*-value > 0.05, and the level of significance is not reached, indicating that there is no bias. In Egger’s regression intercept, *p*-value > 0.05—the level of significance is not reached, indicating that there is no bias. According to the above four bias tests, this study showed no bias.

### 4.2. Heterogeneity Test

Due to the different research designs of the sample subjects, the different sources of bias, and the inconsistency of research quality, the heterogeneity of the research was considered. Therefore, this study used 2 heterogeneity testing methods, the Q test and the I^2^ test. Table 3 is the result of the heterogeneity test in this study.

The Q test is the weighted sum of squared deviations of the effect size. According to Table 3, *p* < 0.001, Q > df corresponding to the overall sample can be obtained, indicating that the research is significant and heterogeneous.

The statistics, I^2^, reflect the proportion of the heterogeneity part in the total variation of the effect size. Higgins et al. [66] pointed out that the tentative standards for the division of I^2^ are as follows: 25%, 50%, and 75%, which represent the low, medium, and high degrees of heterogeneity, respectively. The I^2^ corresponding to the overall sample in Table 3 is 93.878%, which means that the research sample is highly heterogeneous. τ² reflects the true size of the study interval. The meta-analysis results showed that τ² is close to 0, indicating that the true range of the effect size is small [65].

When there was heterogeneity, this study used a random effects model for analysis [65]. When faced with different research objects, the measurement results will be different. 

### 4.3. Outlier Test

**Figure 4:**
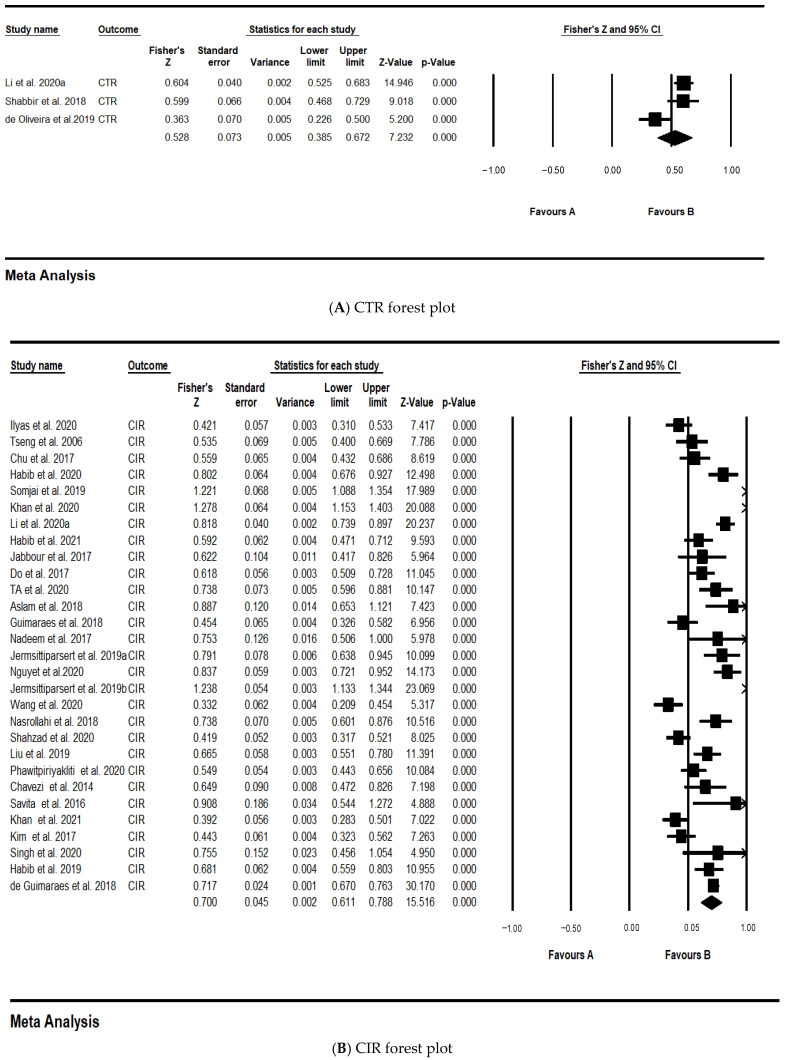

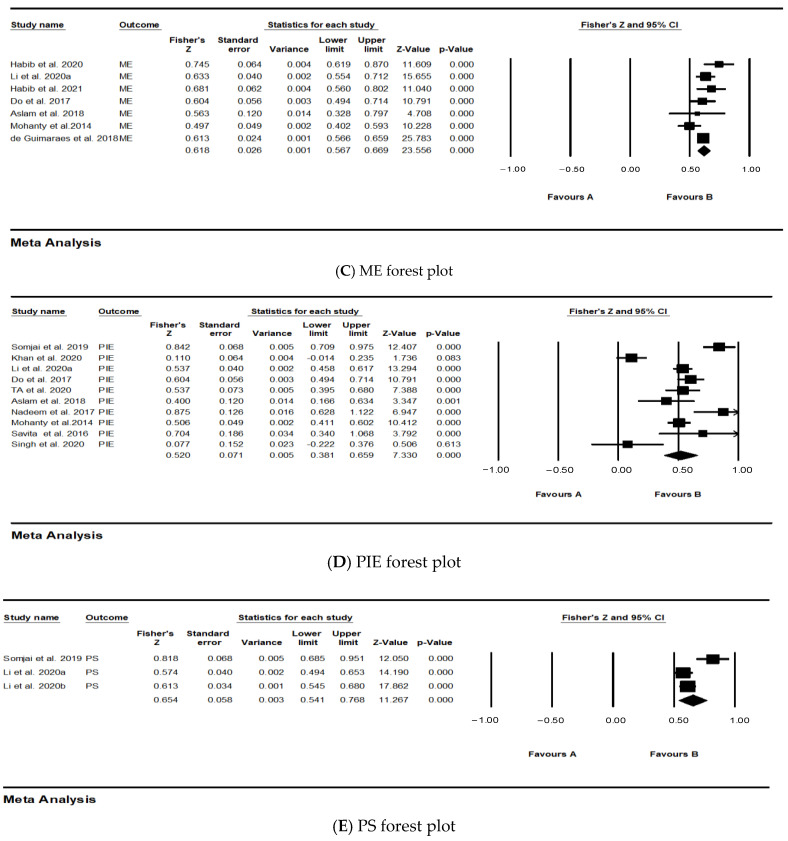
Forest plots.

**Figure 5:**
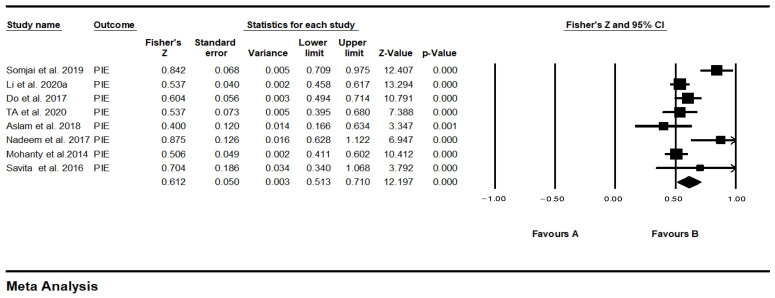
PIE forest plot after removing outliers.

The forest plot can reflect the effect of each study and the 95% confidence interval. In Figure 4, the PIE forest plot, the effective values of Singh et al. [23] and Khan et al. [42] crossed the zero-dividing line at the 95% confidence interval, namely, the 2 outliers of this study. To improve the accuracy of this study, these two samples were deleted, and the PIE forest plot after the deletion is shown in Figure 5. 

### 4.4. Sensitivity Analysis

To identify studies with abnormal effect sizes, this study required a sensitivity analysis to determine if the effect size was abnormal by excluding one target study at a time.

To identify studies with abnormal effect sizes, the research required a sensitivity analysis to determine if the effect size was abnormal by excluding one included study at a time.

In the 3 studies that deleted the CTR one by one, Fisher’s Z changed from 0.528 to 0.603, and I^2^ changed from 78.841% to 0, so the effect size corresponding to the study of de Oliveira et al. [64] is abnormal and should be eliminated. In the 29 studies that deleted the CIR one by one, it was found that the effect size and heterogeneity have not changed significantly, indicating that the research results are relatively stable. In the 7 studies that deleted the ME one by one, Fisher’s Z changed from 0.618 to 0.630, and I^2^ changed from 47.909% to 29.743%, so the effect size corresponding to the study of Mohanty et al. [53] is abnormal and should be eliminated. In the 7 studies that deleted the PIE one by one, Fisher’s Z changed from 0.612 to 0.562, I^2^ changed from 74.103% to 44.408%, so the PIE effect size corresponding to the study of Somjai et al. [41] is abnormal and should be eliminated. In the 3 studies that deleted the PS one by one, Fisher’s Z changed from 0.625 to 0.596, I^2^ changed from 79.659% to 0, so the PS effect size corresponds to the study of Somjai et al. [41] is abnormal and should be eliminated.

### 4.5. Main Effect Test

Table 4 is the meta-analysis results of EGBD and the five dimensions of influencing factors. After excluding the heterogeneous and unqualified studies, there were 2 studies in the target literature that reported the correlation coefficient between CTR and EGBD. The results of random effects model analysis showed the effective degree of CTR and EGBD, Fisher’s Z = 0.603 (*p* < 0.001). A total of 29 studies reported the effect size of CIR and EGBD, Fisher’s Z = 0.700 (*p* < 0.001). A total of 6 studies reported the effect size of ME and EGBD, Fisher’s Z = 0.630 (*p* < 0.001). A total of 7 studies reported the effect size of PIE and EGBD, Fisher’s Z = 0.562 (*p* < 0.001). A total of 2 studies reported the effect size of PS and EGBD, Fisher’s Z = 0.596 (*p* < 0.001). The results of the meta-analysis showed that there was a significant correlation between EGBD and the five influencing factors in the meta-analysis results. Therefore, H1–H5 are all supported.

### 4.6. Moderating Effect Test

This study analyzes the moderators of enterprise size, region, and industry on the effect size, as shown in Table 5. Enterprise size was divided into three groups—ALL, S&M and L. ALL includes micro, small, medium, and large enterprises. S&M includes micro, small, and medium enterprises. L represents large enterprises. Among them, S&M corresponds to the effect size of 0.706 is greater than that of L and ALL, and the moderate effect is more significant and statistically significant (*p* < 0.001). Therefore, H6 is supported. Regions also have a moderating effect. Thailand’s corresponding effect size of 0.914 is greater than that of other regions, and the moderating effect is significant. The conclusion supports H7. 

Among the industries included in this study, the effect size of the automobile manufacturing industry (1.012) and textile industry (0.698) were both larger than the overall effect size. Therefore, H8 was supported.

## 5. Discussion

The research results show that hypotheses H1–H5 are all established, indicating that EGDB is affected by the corporate intangible resources, corporate tangible resources, public supervision, policy and institutional environment, and market environment. Moreover, each item has had a significant positive impact on EGDB. Li et al. [9] verified the results consistent with this study by constructing a least squares structural equation model. In this regard, this study further explores the results of the study by comparing the intensity of each factor.

Research shows that the degree of influence of internal driving factors (0.693) is greater than that of external driving factors (0.602). Possible reasons for this situation follow. From the perspective of internal factors, corporate resources are closely linked to the enterprise. Compared with stereotypes, profit-seeking behavior is the most important factor for enterprises to implement various strategies. For example, as one of the developing countries, China uses its low-cost competitive advantage as the main weapon for Chinese manufacturing to participate in global competition [67]. External factors act on the enterprise in the form of pressure, causing the enterprise to choose different levels of compliance in consideration of pressure. Zhao [29] obtained, by analyzing panel data, that government regulations and public participation in environmental protection can improve the compliance level of enterprises. The effects of various external influence factors have slight differences, but they are all important positive factors. Therefore, this study argues that there is still a need to focus on such external drivers.

There are different degrees of influence among the factors that influence the green development behavior of various enterprises. Research shows that the degree of influence of enterprise intangible resource factors (0.700) is significantly greater than the other 4 factors. This is consistent with the results of other researchers [68]. The possible reasons for this are the following: (1) excessive dependence on traditional material resources can no longer provide a clear competitive advantage [22]; (2) intangible assets, as the foundation of enterprise resources [69], are directly related to enterprise development. Therefore, to better realize EGDB, enterprises need to start from the organizational level. For example, improve the enterprise’s top-level design and formulate a clear enterprise green development strategy around organizational behavior.

In addition, the research results show that the size of an enterprise can adjust its green development behavior. Compared with smaller enterprises, large enterprises are unwilling to take the initiative to change due to the smaller risk they face [70,71]. Therefore, the moderating role of small, medium, and micro enterprises is more obvious. In the process of collecting samples, the researchers found that most of the countries studied for green supply chain management practices and cleaner production behaviors are developing countries, of which the Asian region accounts for a larger proportion. This is related to the earlier attention paid to environmental issues in developed countries, and it has been very mature in the field of green supply chain management practices and cleaner production behaviors. Many enterprises in developed countries choose to use the role of multinational enterprises to transfer product manufacturing to developing countries [72].

In the manufacturing process of enterprises, due to the lack of attention to the environment in the early stage, many environmental pollution incidents have occurred. This also makes the government and society pay more attention to EGDB. From an internal perspective, the corresponding levels of attention and support from different countries and regions will also play a moderating role in the implementation of EGDB. Externally, multinational enterprises have to cater to the needs of target countries and regions and comply with local policies and the wishes of the people. The study found that a total of 28 target studies used research objects covering all walks of life. In addition, different industries have different regulatory effects. Compared with other industries, the automobile manufacturing industry has the greatest impact. The automobile manufacturing process is complex, and many links are full of the possibility of environmental pollution. China’s ecological environment department even promulgated the “Technical Guidelines for Pollution Source Intensity Accounting-Automobile Manufacturing” for the ecological environment standard for the automobile manufacturing industry.

Although this research provides a new perspective of meta-analysis for EGDB, it is also inevitable that some limitations cannot be avoided, like most studies. In the process of data retrieval, to comply with the requirements of the meta-analysis method, it was necessary to use the literature reporting the relevant effect size as the target sample. Therefore, the research on the reported effect size mainly focuses on the literature using the structural equation modeling method. Filtering for a single research method may lead to the omission of related research. Furthermore, despite a large number of searches in multiple databases, empirical research related to public supervision and corporate intangible resources is not yet abundant, resulting in high heterogeneity of research. Future studies can conduct more empirical research on the green development behavior of enterprises based on specific industry backgrounds and pass more industry tests for the research on the green development behavior of enterprises.

## 6. Conclusions

Based on the five dimensions that influence the green development of industrial enterprises divided by GDBP–IE theory, this study constructed a model of the influencing factors of EGDB. Through meta-analysis of 33 empirical studies published in important global academic journals from 2006 to 2021. The meta-analysis method validated the model. In addition, this study explored potential moderator variables, in order to obtain more comprehensive and credible conclusions than similar studies. 

The study found the following:

(1) Corporate intangible resources, corporate tangible resources, public supervision, policy and institutional environment, and market environment all play a positive role in enterprise green development behavior.
(2) Corporate intangible resources have the most significant positive impact on enterprise green development behavior.
(3) The enterprise size, region, and industry all play a regulatory role in EGDB.

From the perspective of theoretical contribution, this research enriches the research field of GDBP–IE theory. The GDBP–IE theory was originally based on industrial enterprises as a starting point to discuss green development behaviors to improve the performance of industrial enterprises. Based on the GDBP–IE theory, this research discusses its influencing factors and applicability in multiple industries, including manufacturing and service industries, and the research conclusions can be applied to relevant research on corporate green development behavior. In this regard, this study uses meta-analysis to systematically integrate the relevant research on EGDB to provide a new understanding of the GDBP–IE theory. In addition, organizational behavior relies on scientific research methods to solve practical problems, and the essence of EGDB still belongs to organizational behavior. Therefore, this research expands the research on EGDB, enriches relevant research on organizational behavior, and provides a basis for researchers to further study EGDB for specific industries.

The contributions of this research to the enlightenment of management practices are as follows: the conclusion proves that improving the level of enterprise green development is inseparable from the efforts of society, enterprises, and the government. To improve the level of green development of an enterprise, it is necessary to expand from the inside to the outside, with the inside of the enterprise as the core. The influence of internal factors of the enterprise is large, so it is necessary to actively utilize the internal resources of the enterprise. By actively creating an atmosphere of green development awareness, enterprise managers structurally influence all levels of enterprise organizational structure, thereby exerting their subjective initiative and regulating corporate tangible resources and corporate intangible resources. The development of an enterprise’s strategy should be based on intangible resources and supplemented by tangible resources. Enterprises actively start from organizational structure, brand reputation, enterprise culture, technological innovation, etc., formulating relevant EGDB, and improving employees’ various levels of behavior—including willingness and abilities on a personal level. Besides, enterprises actively respond to the calls of policies and systems, establish an environmentally friendly social image of the enterprise, make the strategy transparent to the public supervision path, actively use green enterprise strategies to enhance their competitiveness in the market, and value their size, industry, and region. Advantages in these areas thereby enhance the level of green development of the enterprise. The public strengthens the supervision of EGDB, the government attaches importance to the policy and institutional environment, and the market environment is constantly in line with the green development atmosphere, which can positively promote EGDB. Under the influence of the market environment and public supervision, consumers, cooperative enterprises, and competitive enterprises will continue to force enterprises to face a green transformation—the fittest will continue to survive. At the same time, the government provides reasonable rewards and subsidies for EGDB, and strictly punishes enterprises for non-green development behaviors. A series of combined rewards and punishment policies and an institutional environment will promote the formation of a benign ecological environment for EGDB. In addition, according to the size, region, and industry of a given enterprise, it is possible to adjust EGDB. Therefore, the process of implementing EGDB needs to consider the characteristics of its enterprise.

## Figures and Tables

**Table 1:** Encoding table.

	Authors Year ^1^	Outcome	Sample Size	Fisher’s Z	SE	Enterprise Size ^2^	Region	Industry ^3^
1	Ilyas et al. 2020 [24]	CIR	313	0.421	0.057	S&M	Pakistan	Various
2	Tseng et al. 2006 [38]	CIR	215	0.535	0.069	unspecified	China	Electronic Manufacture industry
3	Chu et al. 2017 [39]	CIR	241	0.559	0.065	unspecified	South Korea	Purchase industry
4	Habib et al. 2020 [40]	CIR, ME	246	0.802, 0.745	0.064	L	Bangladesh	Textile manufacture industry
5	Somjai et al. 2019 [41]	CIR, PIE, PS	220	1.221, 0.842, 0.818	0.068	unspecified	Thailand	Car manufacture industry
6	Khan et al. 2020 [42]	CIR, PIE	250	0.856, 0.110	0.064	unspecified	China	Various
7	Li et al. 2020a [9]	CIR, CTR, ME, PIE, PS	615	0.818, 0.604, 0.633, 0.537, 0.573	0.040	unspecified	China	Various
8	Habib et al. 2021 [43]	CIR, ME	266	0.592, 0.681	0.062	unspecified	Bangladesh	Textile manufacture industry
9	Jabbour et al. 2017 [44]	CIR	95	0.622	0.104	ALL	Brazil	Various
10	Do et al. 2017 [45]	CIR, ME, PIE	322	0.618, 0.604, 0.604	0.056	ALL	Vietnam	Various
11	TA et al. 2020 [46]	CIR, PIE	192	0.738, 0.537	0.073	ALL	Vietnam	Various
12	Aslam et al. 2018 [47]	CIR, ME, PIE	73	0.887, 0.563, 0.400	0.120	L	Developing country	Various
13	Guimaraes et al. 2018 [48]	CIR	238	0.454	0.065	ALL	Brazil	Processing manufacture industry
14	Nadeem et al. 2017 [49]	CIR, PIE	66	0.753, 0.875	0.126	ALL	Pakistan	Various
15	Jermsittiparsert et al. 2019a [50]	CIR	166	0.791	0.078	unspecified	Thailand	Electronic manufacture industry
16	Nguyet et al. 2020 [51]	CIR	290	0.837	0.059	ALL	Vietnam	Unspecified
17	Jermsittiparsert et al. 2019b [52]	CIR	350	1.238	0.054	ALL	Thailand	Car manufacture industry
18	Mohanty et al.2014 [53]	ME, PIE	426	0.497, 0.506	0.049	S&M	India	Various
19	Wang et al. 2020 [54]	CIR	260	0.332	0.062	unspecified	China	Various
20	Nasrollahi et al. 2018 [55]	CIR	206	0.738	0.070	unspecified	Iran	Various
21	Shabbir et al. 2018 [56]	CTR	230	0.599	0.066	unspecified	Pakistan	Various
22	Shahzad et al. 2020 [16]	CIR	370	0.419	0.052	unspecified	Pakistan	Service industry
23	Liu et al. 2020 [57]	CIR	296	0.665	0.058	unspecified	China	Various
24	Phawitpiriyakliti et al. 2020 [58]	CIR	340	0.549	0.054	unspecified	Thailand	Pharmaceutical industry
25	Chavezi et al. 2014 [59]	CIR	126	0.649	0.090	L	China	Car manufacture industry
26	Savita et al. 2016 [30]	CIR, PIE	32	0.908, 0.704	0.186	unspecified	Malaysia	Unspecified
27	Khan et al. 2021 [60]	CIR	324	0.392	0.056	S&M	Pakistan	Various
28	Kim et al. 2017 [61]	CIR	272	0.443	0.061	unspecified	South Korea	Unspecified
29	Singh et al. 2020 [23]	CIR, PIE	46	0.755, 0.077	0.152	unspecified	India	Processing manufacture industry
30	Habib et al. 2019 [62]	CIR	262	0.681	0.062	L	Bangladesh	Textile manufacture industry
31	de Guimarães et al. 2018 [63]	ME, CIR	1774	0.717, 0.613	0.024	S&M	Brazil	Various
32	de Oliveira et al.2019 [64]	CTR	208	0.363	0.070	ALL	Brazil	Unspecified
33	Li et al. 2020b [8]	PS	853	0.613	0.034	ALL	China	Various

^1^ To reduce the length, only the first author is listed; ^2^ S&M—medium, small, and micro enterprises; L—large enterprises; ALL—large, medium, small, and micro enterprises; ^3^ various—research objects included multiple industries.

**Table 2:** The publication bias test of 5 factors.

Outcome	Rosenthal’s Fail-Safe N			Begg and Mazumdar Rank Correlation *p*-Value	Egger’s Regression Intercept (2 Tailed)		
	z-Value	*p*-Value	α		*p*-Value	LL ^1^	UL ^2^
CIR	58.839	<0.001	0.05	0.329 (2 tailed)	0.98	−4.009	4.109
CTR	16.838	<0.001	0.05	0.117 (2 tailed)	0.532	−70.479	61.127
ME	33.847	<0.001	0.05	0.652 (2 tailed)	0.811	−3.083	3.754
PIE	24.175	<0.001	0.05	0.322 (2 tailed)	0.388	−2.62	5.84
PS	25.463	<0.001	0.05	0.602 (2 tailed)	0.319	−37.148	49.594

^1,2^ LL and UL indicate the lower limit and upper limit, respectively, of the 95% confidence interval of Egger’s regression intercept.

**Table 3:** The heterogeneity test of the whole sample.

Model	k	Combined Effect Size	95%CI		Q-Value	df	*p*-Value	I^2^	τ^2^
			LL	UL					
fixed	50	0.648	0.633	0.664	537.140	49.000	<0.001	90.878	0.030
random	50	0.661	0.609	0.714					

Note: CI—confidence interval.

**Table 4:** EGBD and five dimensions of influencing factors of the meta-analysis results.

Outcome	k	Effect Size	95%CI		*p*-Value
			LL	UL	
CTR	2	0.603	0.535	0.670	<0.001
CIR	29	0.700	0.611	0.788	<0.001
ME	6	0.630	0.596	0.664	<0.001
PIE	7	0.562	0.491	0.634	<0.001
PS	2	0.596	0.545	0.648	<0.001

**Table 5:** Moderator’s analysis results.

Outcome	Type	k	Fisher’s Z	95% CI		Q-Value	df	*p*-Value	I^2^	τ^2^	Qb
				LL	UL						
Enterprise size	ALL	18	0.657	0.632	0.681	196.480	17	<0.001	91.348	0.030	16.112
	S&M	7	0.706	0.647	0.766	13.443	6	<0.05	55.366	0.008	
	L	6	0.605	0.577	0.632	56.362	5	<0.001	91.129	0.014	
Region	Thailand	6	0.914	0.863	0.965	107.394	5	<0.001	95.344	0.083	537.140
	Malaysia	2	0.806	0.549	1.063	0.600	1	>0.05	0.000	0.000	
	Iran	1	0.738	0.601	0.876	0.000	0	>0.05	0.000	0.000	
	Bangladesh	5	0.698	0.643	0.753	6.282	4	>0.05	36.322	0.002	
	Vietnam	6	0.656	0.607	0.705	15.460	5	<0.05	67.659	0.008	
	China	11	0.644	0.616	0.672	157.493	10	<0.001	93.651	0.033	
	Brazil	5	0.636	0.606	0.667	35.642	4	<0.001	88.777	0.013	
	Developing country	3	0.617	0.481	0.752	8.611	2	<0.05	76.773	0.047	
	India	3	0.514	0.448	0.580	2.637	2	>0.05	24.144	0.001	
	South Korea	2	0.497	0.410	0.584	1.694	1	>0.05	40.983	0.003	
	Pakistan	6	0.480	0.427	0.533	22.658	5	<0.001	77.932	0.017	
Industry	Car manufacture industry	5	1.012	0.954	1.071	57.905	4	<0.001	93.092	0.062	190.159
	Electronic manufacture industry	2	0.646	0.545	0.747	6.053	1	<0.05	83.480	0.027	
	Pharmaceutical industry	1	0.549	0.443	0.656	0.000	0	>0.05	0.000	0.000	
	Processing manufacture industry	2	0.500	0.383	0.618	3.294	1	>0.05	69.638	0.032	
	Purchase industry	1	0.559	0.432	0.686	0.000	0	>0.05	0.000	0.000	
	Service industry	1	0.419	0.317	0.521	0.000	0	>0.05	0.000	0.000	
	Textile manufacture industry	5	0.698	0.643	0.753	6.282	4	>0.05	36.322	0.002	
	Various	28	0.628	0.610	0.646	236.329	27	<0.001	88.575	0.019	

## Data Availability

Not applicable.
